# Nutrition Genomics

**DOI:** 10.3390/ijms24076490

**Published:** 2023-03-30

**Authors:** Jesús Osada

**Affiliations:** 1 Departamento de Bioquímica y Biología Molecular y Celular, Facultad de Veterinaria, Instituto de Investigación Sanitaria de Aragón, Universidad de Zaragoza, E-50013 Zaragoza, Spain; josada@unizar.es; 2 Instituto Agroalimentario de Aragón, CITA-Universidad de Zaragoza, E-50013 Zaragoza, Spain; 3 CIBER de Fisiopatología de la Obesidad y Nutrición, Instituto de Salud Carlos III, E-28029 Madrid, Spain

This Special Issue is devoted to nutrition genomics, which is the characterization of the whole genome response to nutrients, in an effort to gather all the available pertinent information and to establish the foundation for a future encyclopedia of genomic responses driven by diets or nutrients.

Regarding this point, one of the manuscripts submitted to this Special Issue provides evidence that the administration of erythrodiol, a terpenic compound found in a large number of plants, significantly modified the mouse hepatic transcriptome, specifically, the expression of the genes involved in detoxification processes, protein metabolism, and nucleic-acid-related metabolites. This induced hepatic transcriptional change was selective in terms of sex and required a threshold dose [1]. Additionally in mice, the effects of high-fat diets containing palm oil, hybrid palm oil, or olive oil were studied at the transcriptome level in the liver. The olive oil group showed changes in the genes related to amino acid and fatty acid metabolisms, the palm oil group showed changes related to calcium ion homeostasis, and the hybrid palm oil group showed changes in relation to protein localization. The latter oils induced non-alcoholic steatohepatitis in mice via altered hepatocyte transcription [2]. In this way, both pieces of research offer a glimpse of methods that can be utilized to understand the highly prevalent non-alcoholic fatty liver disease and potential diets or nutrients to treat the disease.

The effect of a single nutrient, in this case one dose of cholecalciferol (1000 IU), on whole blood gene expression levels was explored in 22 adults. The results showed that this vitamin modulated the expression of the solute carrier family 25 member 20, the gene involved in fatty acid oxidation, various transcription factors, and the genes related to glucose metabolism [3]. 

The influence of fat content on brown adipose tissue thermogenesis was explored regarding its function in the presence or absence of apolipoprotein A-IV. This apolipoprotein modulated the response to this type of diet regarding the uncoupling of protein 1 and the expression of thermogenic genes [4]. This exemplifies the influence of certain proteins on the response of nutrients on gene expression in certain tissues.

Mammary glands have been the focus of several studies assessing the influence of dietary changes in fat or global nutrients. Ammah et al. [5] and Ibeagha-Awemu et al. 2018 [6] are complementary studies that analyzed the mammary glands of cows following their dietary supplementation with 5% linseed oil or safflower oil for 28 days. In the first study, which analyzed the miRNA transcriptome, the authors found three hub miRNAs—miR-30d, miR-484, and miR-16b—connected to cell cycle arrest, p53 signaling, and transforming growth factor-beta that have a considerable influence on milk and blood phenotypes [5]. In the second study, long non-coding RNA expression was characterized. The authors found 4630 novel long non-coding RNAs. Fifty-three lncRNAs were differentially expressed by the dietary intervention which could modulate the response to these dietary components [6]. Both studies have paved the way to understanding the role of non-translated RNAs and their involvement in gene expression in certain tissues. In a third study carried out in this tissue, the authors investigated the impact of nutrient restriction and its consequences on an inflammatory challenge given by the administration of lipopolysaccharide. In this experimental setting, dietary restriction had a profound impact on the beta-oxidation process, which may affect the response to inflammation [7]. This paper offers a perspective on the way that nutritional status may condition other cellular processes such as inflammation by changes in the gene expression of the enzymes involved in lipid metabolism.

Epigenomics has also been represented by the analysis of DNA methylation following the timing of the administration of a high-fat diet. To this end, such diets were given to pregnant mothers or to post-weaning male rats. Genome-wide DNA methylation revealed 1744 differential methylation regions between the groups with the majority of them located outside of gene-coding regions. The insulin and phosphatidylinositol (PI) signaling pathways were particularly enriched in these methylated bases [8]. These results evidenced that the timing of HF diet intake determines DNA methylation of certain gene expressions in hepatic metabolic pathways and provides a new level of complexity in the control of gene expression by nutrients.

Several reviews have explored the way nutrients regulate gene expression. In one study, the control of gene expression was the focus of attention through the actions of three sensors: the carbohydrate-responsive element binding protein for sugars, the peroxisome proliferator-activated receptors for fat, and the mammalian target of the rapamycin complex 1 which responds to amino acid concentrations [9]. A second study addressed a more specific aspect, for example, protein post-translational modification through palmitoylation, which may influence chromatin remodeling enzymes, transcription factors, and nuclear proteins [10]. A third study explored the regulation of DNA methylation executed by DNA methyltransferases (DNMT1, DNMT3a, and DNMT3b) which require S-adenosylmethionine. The modulation of these enzymatic reactions by polyamines may have profound consequences on the gene methylation of genes and their expression [11]. The fourth paper addresses the impact of trimethylamine (TMA) and its concomitant conversion into trimethylamine-N-oxide (TMAO) by liver flavin monooxygenase 3 (FMO3). TMAO promotes foam cell formation, deregulating enterohepatic cholesterol and bile acid metabolism and impairing macrophage reverse cholesterol transport. In addition, FMO3 promotes dyslipidemia by regulating the genes involved in hepatic lipogenesis, gluconeogenesis, and cholesterol homeostasis. Both TMAO and FMO3 seem to have independent effects on lipid metabolism and atherogenesis [12]. What is more interesting is the fact that TMAO is produced by intestinal microbiota providing in this way a new level of complexity of the world of nutrients and gene expression by the metabolites of the former generated by metagenomics. The final paper reviewed the genetic basis of obesity and body fat distribution providing further evidence of the interactions among genetic variations and environmental factors (the abundance of palatable, energy-dense diets, reduced physical activity, and increased sedentary lifestyle) [13]. A complete understanding of these mechanisms will improve our knowledge of metabolic diseases such as obesity, insulin resistance, type 2 diabetes, and cardiovascular disease. Furthermore, this knowledge will foster new therapeutic approaches based on nutrition and genetic backgrounds.

In conclusion, the above manuscripts offer a new perspective on the complexity of the impact of diet and nutrient-dependent signals. This aspect has to be addressed on an organ basis and is influenced by genetic make-up defined by protein variants and epigenomics marks and other environmental influences where metagenomics plays an important role. This complexity and the availability of data through Genome Expressed Omnibus (https://www.ncbi.nlm.nih.gov/geo/ (accessed on 18 March 2023)) offer a fertile ground for generative artificial intelligence to find patterns that are able to be categorized and to forecast the crucial bottlenecks causing diseases.
